# Supplementary material for: Effectiveness and Safety of Ayurvedic Medicines in Type 2 Diabetes Mellitus Management: A Systematic Review and Meta-Analysis
Source: Front Pharmacol. 2022 Jun 8;13:821810. doi: 10.3389/fphar.2022.821810 (PMC9213670; doi:10.3389/fphar.2022.821810)
Supplement: Supplementary file 1 [file DataSheet1.zip › Supplementary Material/Forest and Funnel Plots/Nigella sativa/Insulin resistance.pdf]

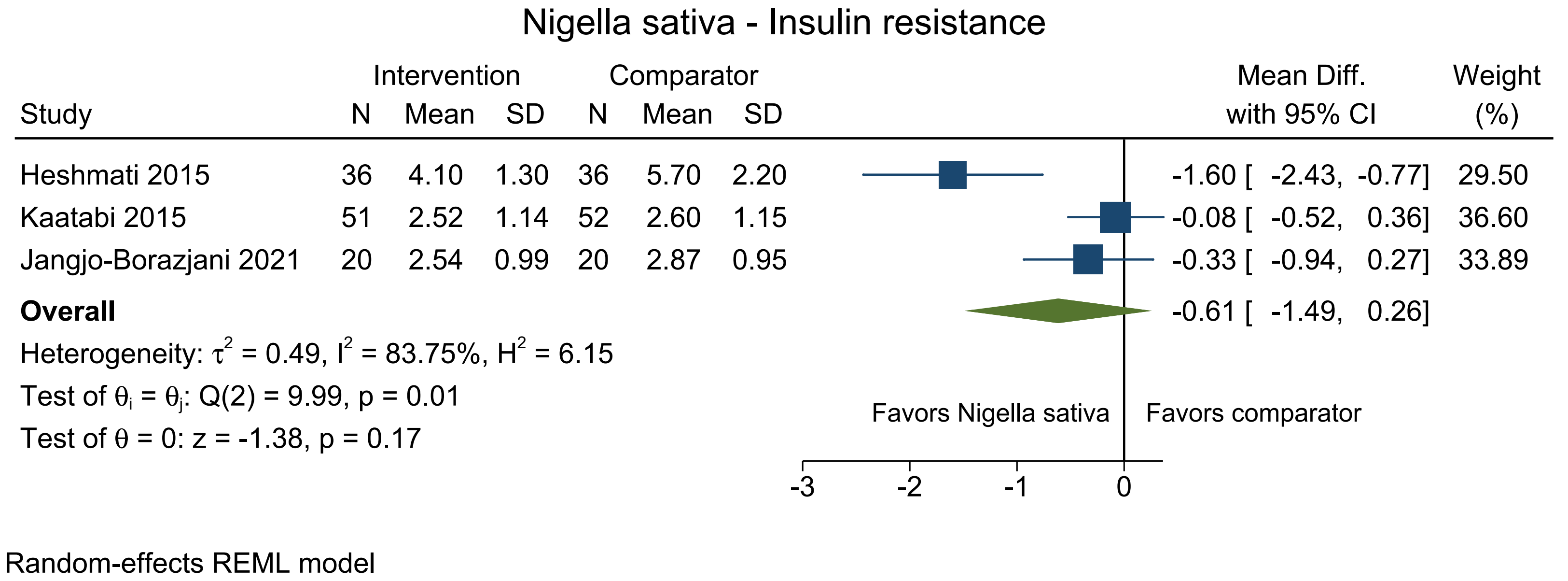
Nigella sativa - Insulin resistance
Intervention
Comparator
Study
N
Mean
SD
N
Mean
SD
Mean Diff.
with 95% CI
Weight
(%)
Heshmati 2015 36 4.10 1.30 36 5.70 2.20 -1.60 [-2.43, -0.77] 29.50
Kaatabi 2015 51 2.52 1.14 52 2.60 1.15 -0.08 [-0.52, 0.36] 36.60
Jangjo-Borazjani 2021 20 2.54 0.99 20 2.87 0.95 -0.33 [-0.94, 0.27] 33.89
Overall -0.61 [-1.49, 0.26]
Heterogeneity: τ^2 = 0.49, I^2 = 83.75%, H^2 = 6.15
Test of $\theta_i = \theta_j$: Q(2) = 9.99, p = 0.01
Test of θ = 0: z = -1.38, p = 0.17
Favors Nigella sativa
Favors comparator
-3
-2
-1
0
Random-effects REML model
